# Synthesis, Characterization and Catalytic/Antimicrobial Activities of Some Transition Metal Complexes Derived from 2-Floro-N-((2-Hydroxyphenyl)Methylene)Benzohydrazide

**DOI:** 10.3390/molecules29235758

**Published:** 2024-12-05

**Authors:** Ahmed K. Hijazi, Ziyad A. Taha, Dua’a K. Issa, Heba M. Alshare, Waleed M. Al-Momani, Ali Elrashidi, Ahmad S. Barham

**Affiliations:** 1 Department of Chemistry, College of Arts and Sciences, University of Petra, P.O. Box 961343, Amman 11196, Jordan; 2 Department of Chemical Sciences, Faculty of Science and Arts, Jordan University of Science and Technology, P.O. Box 3030, Irbid 22110, Jordan; tahaz33@just.edu.jo (Z.A.T.); dkissa20@sci.just.edu.jo (D.K.I.); hmalshare@just.edu.jo (H.M.A.); 3 Department of Basic Medical Sciences, Faculty of Medicine, Yarmouk University, Irbid 21163, Jordan; waleed.momani@yu.edu.jo; 4 Electrical Engineering Department, College of Engineering, University of Business and Technology, Jeddah 23435, Saudi Arabia; a.elrashidi@ubt.edu.sa; 5 Engineering Mathematics Department, Faculty of Engineering, Alexandria University, Alexandria 21544, Egypt; 6 Department of Chemistry, School of Science, The University of Jordan, Amman 11942, Jordan; a.barham@ju.edu.jo

**Keywords:** aniline oxidation, catalysis, antimicrobial, transition metal complexes

## Abstract

Background: In the last few decades, the field of coordination chemistry has grown very fast, especially in the fields of pharmaceutical, biological and catalytic studies. In ancient times, metals were thought to be beneficial to health issues but nowadays the link between organic–metal substances and different industrial and medicinal properties is well established. Methods: A Schiff base ligand (2-fluoro-N’-[(E)-2-hydroxyphenyl) methylene] benzohydrazide) was reacted with a series of transition metals to produce complexes with a general formula [ML_2_(NO_3_)]NO_3_.nH_2_O, where [M = Zn, Cu, Co, Ni, Mn], and [n = 0, 1], corresponding to complexes **1**–**5**. The nature of the bond was determined in the solid state and solution using spectral studies (^1^H-NMR, ^13^C-NMR, UV-Vis and FT-IR), TGA, EPR, elemental analysis and molar conductivity measurement. Results: All M(II) complexes are 1:1 electrolytes, as illustrated by their molar conductivities. The results demonstrate that all synthesized complexes present a coordination number of six by the bonding of the bidentate ligand via its azomethine nitrogen atoms and carbonyl oxygen atoms, as well as with one nitrate group as a bidentate ligand via two oxygen atoms. The DPPH radical scavenging technique was used to investigate the antioxidant activities of the ligand [L] and the metal complexes. It is clear that the activity increased in M (II) complexes compared to the Schiff base ligand. Complex **5** showed the highest activity, with an excellent activity of 90.4%, while complex 4 showed the lowest. The antibacterial activities of the Schiff base and its complexes have been examined against various pathogenic bacteria to measure their inhibition potential. Complex **2** showed remarkable activity against Gram (+) bacteria and fungi with an MIC value of 8 μg/mL, which is greater than that of the positive controls, oxytetracycline and fluconazole. The catalytic activities of all complexes were examined in the oxidation of aniline, and the results illustrated that all complexes had a 100% selectivity in producing only azobenzene, and complex **4** had the highest activity (91%). Conclusion: The obtained results from this study show that the antioxidant and antibacterial properties of both the Schiff base ligand and its derived complexes are promising, with some demonstrating remarkable activities. Moreover, the catalytic activities and selectivities of the prepared complexes in aniline oxidation are interesting.

## 1. Introduction

There is a critical need to develop novel antimicrobial classes with different chemical structures and mechanisms of action as a result of the major escalation of bacterial pathogens in recent decades. The interaction between metals and Schiff bases via their chelating sites (N, O, and S) plays a crucial role in enhancing the biological activity of various metallobiomolecules. Research indicates that metal ion-interacted Schiff base ligands exhibit enhanced antimicrobial activity compared to free ligands [1,2].

Schiff base compounds gained interest due to their ability to complex with many different transition metals in various coordination modes and oxidation states mainly via nitrogen and oxygen atoms [3,4,5,6,7,8,9,10]. They appear in other structures and have remarkable applications [11,12]. They play a critical role in inorganic chemistry because of their stability in various oxidation states. The primary applications of Schiff bases are in catalysis [13,14,15,16] and in biological fields like antimicrobial, antibacterial, antifungal, antitumor, and antioxidant substances [17,18,19,20]. Furthermore, they possess many analytical and industrial applications [21,22,23,24]. According to the above discussions, we assume that a new Schiff base with a new structure might exhibit novel spectral and biological characteristics [3,4,5,6,7,8,9,10].

In 2023, Awolope et al. investigated the antioxidant and antimicrobial activities of the 4,4′-{ethane-1,2-diylbis[nitrilo(Z)methylylidene]}bis(2-methoxyphenol) ligand with Co(II), Ni(II), Cu(II), ZrO(IV), VO(IV), UO_2_ (II) complexes. All complexes revealed excellent antibacterial and antioxidant activities [25]. Another group studied Schiff base ligands and metal complex formation with Iron (III) and Zinc (II). All metal complexes demonstrated impressive antibacterial and antifungal activities compared to free ligands, especially the Zn (II) complex, and the ligands showed better antioxidant behavior than their metal complexes [26].

Transition metals, including Mn, Fe, Co, Ni, Cu and Zn, play indispensable roles in living organisms, functioning at the molecular level. They form essential components of metalloproteins and are crucial for various biological processes. The coordination chemistry of transition metals, particularly their ability to adopt multiple coordination geometries and oxidation states, is fundamental to their biological functions [27]. El-Sonbati et al. synthesized a new Schiff base ligand (E)-2-(((3-aminophenyl)imino)methyl)phenol with a series of transition metals. It was noted from the spectroscopic data that transition metals coordinate to the ligand through the NH_2_ group, the protonated phenolic oxygen and the azomethine group [28].

Recently, a group of researchers synthesized and investigated a group of biologically active Schiff base ligands and their complexes with transition metals (V, Fe, Co, Ni, Cu, Zn, Zr, Rh, Pd, Cd, Sn, Ir, Pt and Pb) to study the antibacterial, antifungal, anti-viral, antimicrobial, anti-cancer and corrosion-inhibiting activities [29].

Herein, we describe the synthesis of new metal–organic complexes using 2-fluoro-N’-[(E)-2-hydroxyphenyl) methylene] benzohydrazide Schiff base ligands and transition metals. Different spectroscopic techniques characterize the synthesis complexes and investigate their antioxidant, antimicrobial and catalytic properties.

## 2. Results and Discussion

**Scheme 1:**
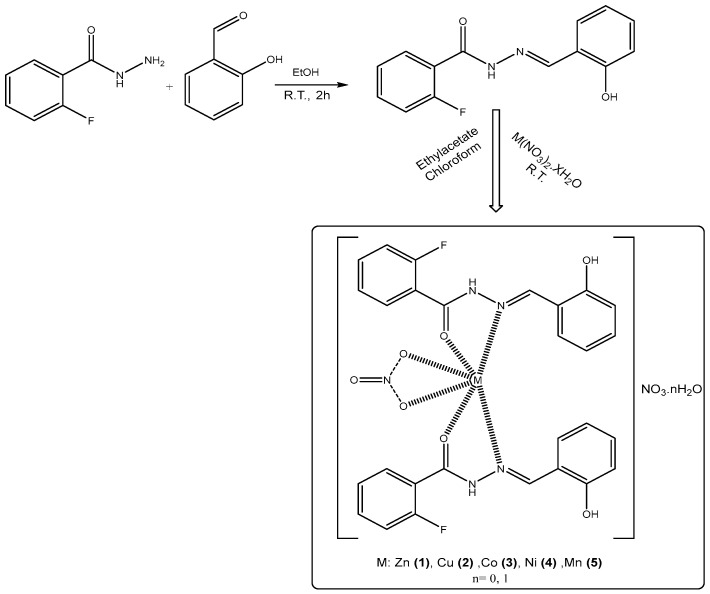
Synthetic route for complexes **1**–**5**.

Bidentate ligand L was prepared by condensing 2-flurobenzoic hydrazide and salicylaldehyde at a 1:1 molar ratio. L is soluble in common organic solvents like toluene, methanol, benzene and ethanol. Metal (II) complexes **1**–**5** present a coordination number of six by the bonding of the bidentate ligand via its azomethine nitrogen atoms and carbonyl oxygen atoms, as well as with one nitrate group as a bidentate ligand via two oxygen atoms. The stoichiometry of the complexes derived from elemental analysis corresponds to the general formula [ML_2_(NO_3_)](NO_3_).n(H_2_O) [M = zinc, copper, cobalt, manganese and nickel], and [n = 0, 1], as presented in Scheme 1. They are non-hygroscopic solids, soluble in DMSO, DMF and methanol. The molar conductivity values presented in Table 1 confirm the 1:1 electrolytic characteristic of all the prepared complexes.

### 2.1. Infrared Spectroscopy

**Figure 1:**
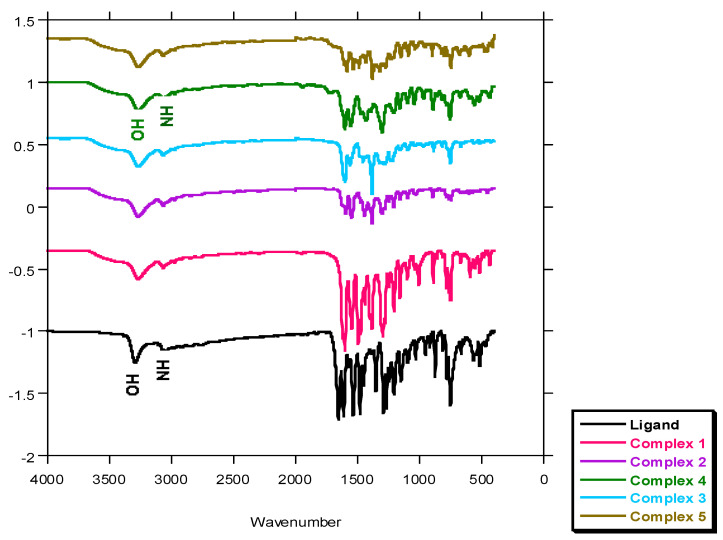

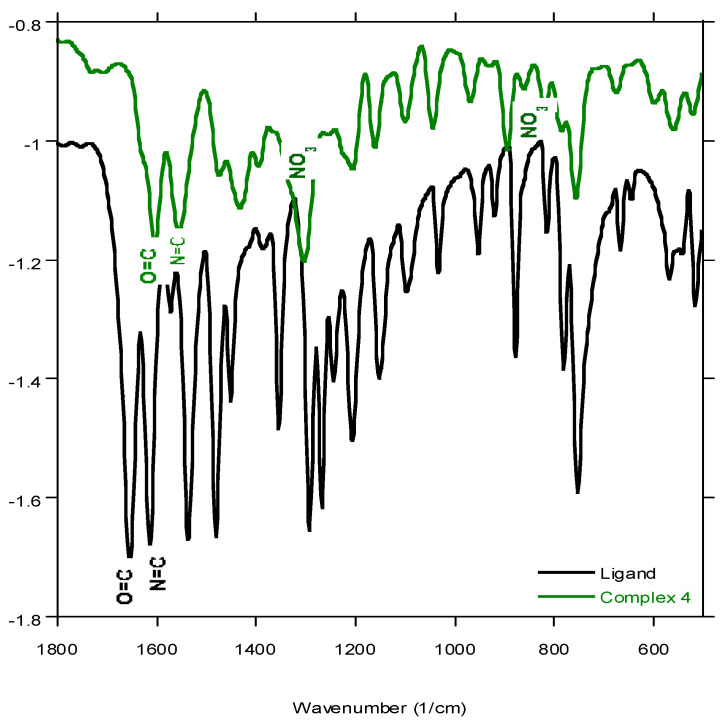
FT-IR spectra of Schiff base ligand and M(II) complexes at different wavenumbers.

The binding mode for the ligand coordinated to the metal ion was investigated by comparing the IR spectrum of the free ligand in the (4000 to 400) cm^−1^ range with the spectra of its complexes, as shown in Table 2. The IR spectra of the five complexes show a shift compared with ligand characteristic bands due to complex formation. The IR spectra of the ligand and its complexes are given in Figure 1. In the IR spectrum of the ligand, the band at 1617 cm^−1^ is referred to as the azomethine group (C=N), while another band at 1660 cm^−1^ is indexed to the C=O group. After complexation, the HC=N and C=O carboxyl moiety absorption is shifted to lower wavenumbers, (1560–1537) and (1608–1600) cm^−1^; respectively. The complexes IR spectra show two new peaks because of oxygen and nitrogen bonding to the metal ion, and the vibrations (M-N) and (M-O) appear in the range of 547–565 cm^−1^ and 418–430 cm^−1^ as medium intensity bands, respectively [30,31,32]. The new bands that appeared in the spectra of all complexes in the range of 1390–1380 cm^−1^ are attributed to the presence of the free (non-coordinated) nitrate group. This result is confirmed by the values of the conductivity of all investigated complexes. The coordinated nitrate group is assigned to bidentate coordination with M(II) ion via two oxygen atoms. The IR spectral data of the free ligand also show vibration bands at 3060 cm^−1^ and 3300 cm^−1^, which are attributed to the –NH group and –OH groups, respectively [33]. After complexation, the positions of these two bands are slightly shifted, confirming the coordination of the ligand to the metal ions.

The IR spectra of the complexes also displayed absorption bands at around 1440–1470, 1300–1333, 1090–1102 and 810–835 cm^−1^, corresponding to the stretching modes of a coordinated nitrate ion, compared to the free ligand. The absorption bands at 1440–1470 and 1300–133 cm^−1^ are assigned to the ν_1_ and ν_4_ stretching modes of the coordinated nitrate groups (C_2v_), respectively [34]. The separation of the two strongest frequencies [ν_1_–ν_4_] of 170–125 cm^−1^ is observed in all complexes, which is consistent with the general pattern observed for nitrate ions coordinated bi-dentately to metals [34,35]

### 2.2. UV–Visible Spectroscopy

**Figure 2:**
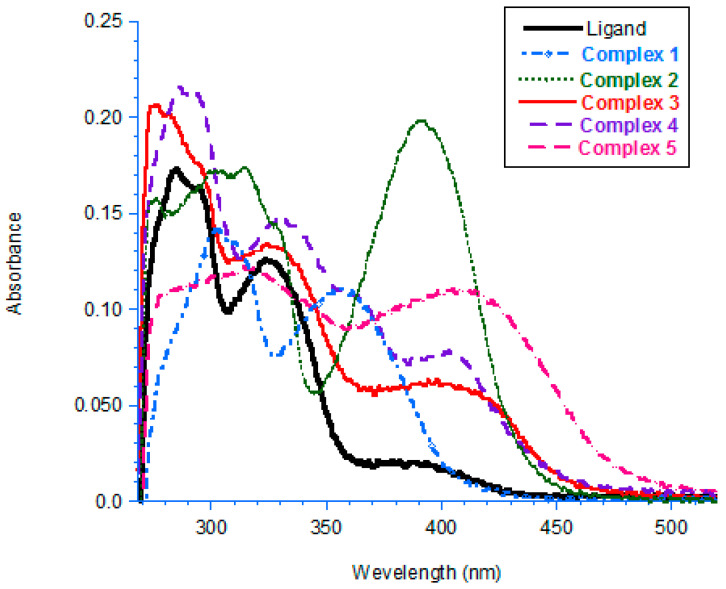
UV–visible spectra of Schiff base ligand and its metal complexes (1 × 10^−5^ M in DMF at 25 °C).

UV-Vis spectroscopy is considered a very useful technique to validate the results provided by other methods of structural investigation. The maximum absorption wavelength (λ_max_) of the ligand L and its M(II) complexes (1 × 10^−5^ M in DMF at 25 °C) are listed in Table 3. Figure 2 shows the absorption spectrum of the ligand **L,** which shows mainly four absorption bands in the region (200–450) nm. The first two high-intensity bands observed at λ_max_ = 284 and 296 nm are associated with the π-π* spin-allowed transitions of the benzene rings (conjugated system). The absorption band at 325.4 nm may correspond to the π-π* transition of the azomethine group C=N. The last band appears at a λ_max_ of 387.7 nm and could be attributed to the n- π* transition of the conjugation between the lone pair of electrons of the p-orbital of the oxygen atom in (C=O) [36]. Figure 2 shows that all complexes had nearly identical absorption bands with a different change in the value of ε depending on the type of the ion. The similarity in the spectra indicates that all M(II) complexes have similar structures. These shifts and changes in the positions of the bands support the coordination of the free ligand to the metal ions via the C=O and HC=N groups. The metal–ligand charge transfer absorption bands are identified in the visible region of all complexes [37].

### 2.3. Thermogravimetric (TG) Analysis

**Figure 3:**
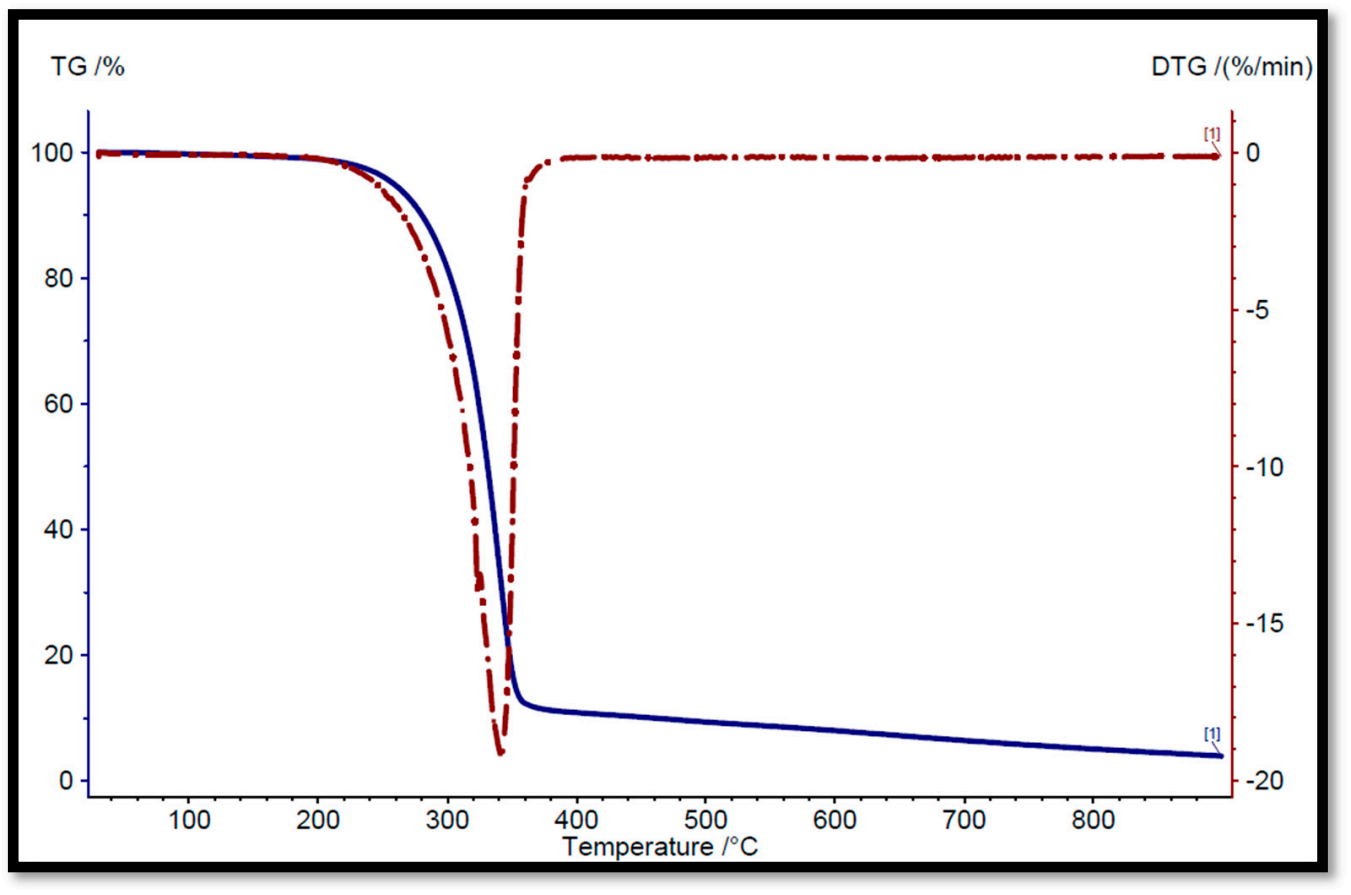
TGA and DTGA of free ligand.

TGA and differential thermal analysis (DTGA) of the Schiff base ligand and its metal complexes were carried out under N_2_ flow at temperatures up to 900 °C. The TGA and DTGA data of the ligand and M(II) complexes are listed in Table 4. The TGA and DTGA curves of the ligand (Figure 3) illustrate that one decomposition step occurs at about 307.7 °C and is finished at 387.0 °C, associated with a mass loss of 87.7 wt%. The remaining weight may correspond to some hydrocarbon ashes. Thermogravimetry plays a crucial role in complex decomposition, which may affect the antimicrobial activity of these complexes.

**Figure 4:**
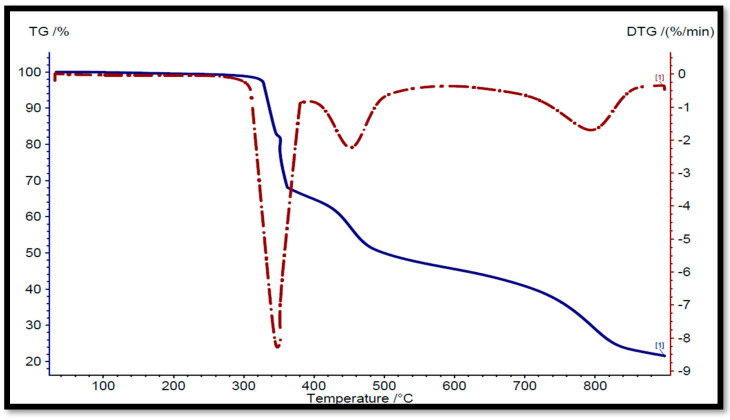
TGA and DTGA of complex **1**.

The TGA-DTG curve of complex **1** shows that the first decomposition step occurs in the range of (319–364) °C, with an onset at 328.3 °C, and is associated with a mass loss of 30.36 wt%. This corresponds to the loss of two NO_3_ and part of the ligand moiety around Zn (II). The second decomposition step in the range of (431–482) °C, with an onset at 440.0 °C, is associated with a mass loss of 9.89 wt.%. The final decomposition of this complex occurs at a range between (763–840) °C, with an onset of 777.7 °C, and is associated with a mass loss of 11.07 wt%. These mass losses in the second and third decomposition stages likely correspond to the complement of the ligand moiety, as shown in Figure 4.

The TGA-DTG curve of complex **2** shows that the first decomposition occurs at a range of (151–219) °C, with an onset at 167.1 °C. It is associated with a mass loss of 8.14 wt%. This mass loss could be attributed to the presence of the uncoordinated nitrate group. The second decomposition stage with a temperature range of (248–374) °C, with an onset at 274.7 °C, is associated with a mass loss of 25.53 wt.%, which likely corresponds to the loss of the coordinated nitro group and the ligand moiety.

The TGA-DTG curve of complex **3** shows four decomposition stages. The first decomposition temperature range (30.74–238) °C, with an onset at 38.9 °C, is associated with a mass loss of 13.85 wt%. This corresponds to losing one molecule of uncoordinated water and the free NO_3_ ion. The second decomposition stage with a temperature range of (238–506) °C, with onset at 299.8 °C, is associated with a mass loss of 38.8 wt.%. This mass loss likely corresponds to the loss of the coordinated nitro group and a part of the ligand moiety. The third decomposition step in the range of (506–680) °C, with an onset at 542.0 °C, has a mass loss of 16.37 wt%, while the fourth decomposition between (680 and 816) °C, with an onset at 733.0, has a mass loss of 8.18 wt%. The total mass losses in the last two steps refer to the complement of the ligand.

The TGA-DTG curves of complex **4** show that the first decomposition temperature range (30.11–137) °C, with onset at 72.0 °C, is associated with a mass loss of 3.29 wt%. This corresponds to losing one molecule of uncoordinated water around Ni(II). The second decomposition stage with a temperature range of (166–314) °C, with an onset at 227.2 °C, is associated with a mass loss of 18.87 wt.%. This mass loss in the second decomposition stage likely corresponds to the loss of two nitro groups. The third, fourth and fifth decomposition steps show a total mass loss of 37.40%. This total mass loss in the last three decomposition steps refers to the decomposition of the ligand moiety.

The TGA-DTG curve of complex **5** shows that the first decomposition stage occurs between (207 and 357) °C, with onset at 271.4 °C, and is associated with a mass loss of 32.89 wt%. This corresponds to the loss of two NO_3_ and part of ligand moiety around Mn (II). The second decomposition step starts from (357 to 537) °C and is associated with a mass loss of 23.04 wt.%. This mass loss likely corresponds to the complement of the ligand moiety.

### 2.4. ^1^H NMR Characterization

**Figure 5:**
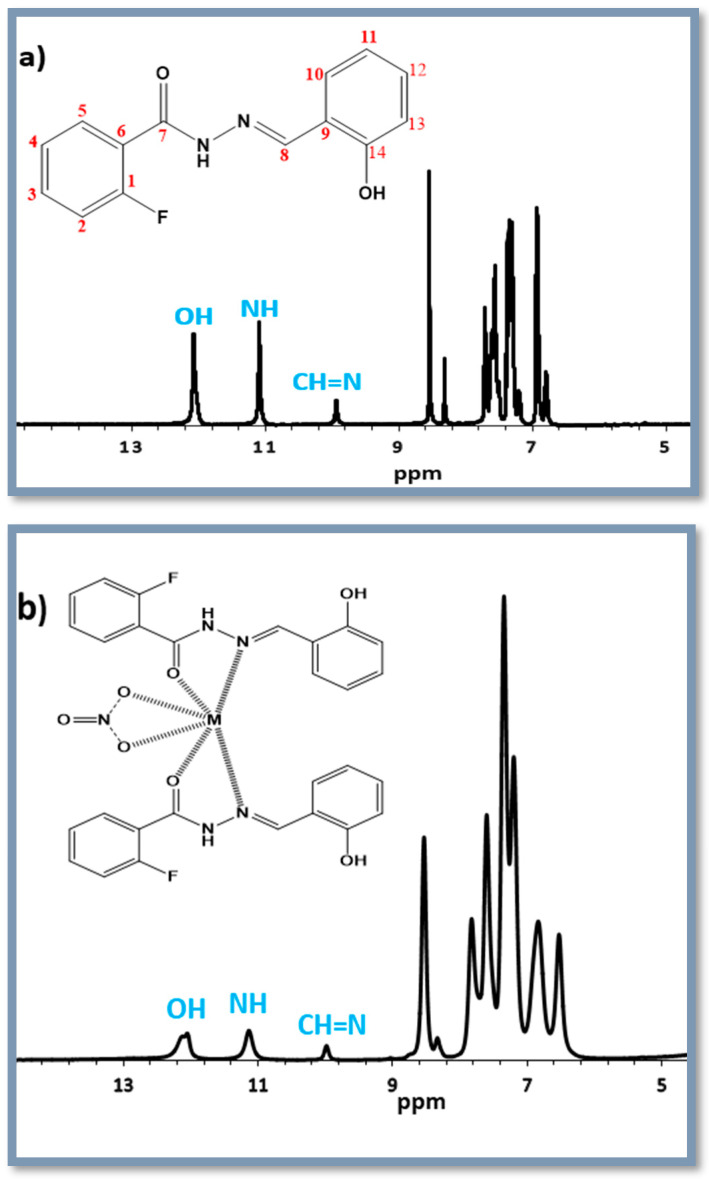
^1^H-NMR Spectra of (**a**) Schiff base ligand (**b**) complex (**1**) in DMSO d_6_.

^1^H NMR spectra of the ligand and complex **1** were recorded in DMSO-d_6_. The characteristic signals appearing at 11.11 and 12.08 ppm in the spectrum of the free ligand are assigned to the protons of the NH and OH groups, respectively. After complexation, the OH and NH peaks are slightly shifted downfield [38]. The azomethine (CH=N) proton of the free ligand appears as a singlet peak observed at δ = 9.92 ppm. This peak is shifted downfield after complexation to δ = 10.10, which confirms the coordination between the ligand and the metal ion. The peaks observed at a range of δ = 6.79–7.73, for ligand L, are assigned to the protons of the aromatics as multiplet peaks (Figure 5a). The spectrum of complex **1** shows that these peaks are slightly shifted or almost unchanged (Figure 5b). It is worth mentioning that complexes **2**–**5** are paramagnetic, so it is difficult to measure NMR for them.

### 2.5. ^13^C NMR Characterization

The ^13^C-NMR spectra of the free ligand and complex **1** were recorded in a deuterated dimethylsulfoxide (DMSO-d_6_) solution. The most important peaks are for the (C=O) and (C=N) groups. The ligand spectrum displayed two signal peaks at 160.20 and 148.33 ppm assigned to carbonyl (C=O) carbon and azomethine carbon (C=N), respectively, as represented in Figure S5 (supplementary information). After complexation, it was observed that different intensity signals for (C=O) and azomethine carbon (C=N) were found to shift downfield to 161.16 and 148.67 ppm, respectively (Figure S6). These results confirm the presence of a bond between zinc–oxygen and zinc–nitrogen atoms.

### 2.6. EPR Measurements

**Figure 6:**
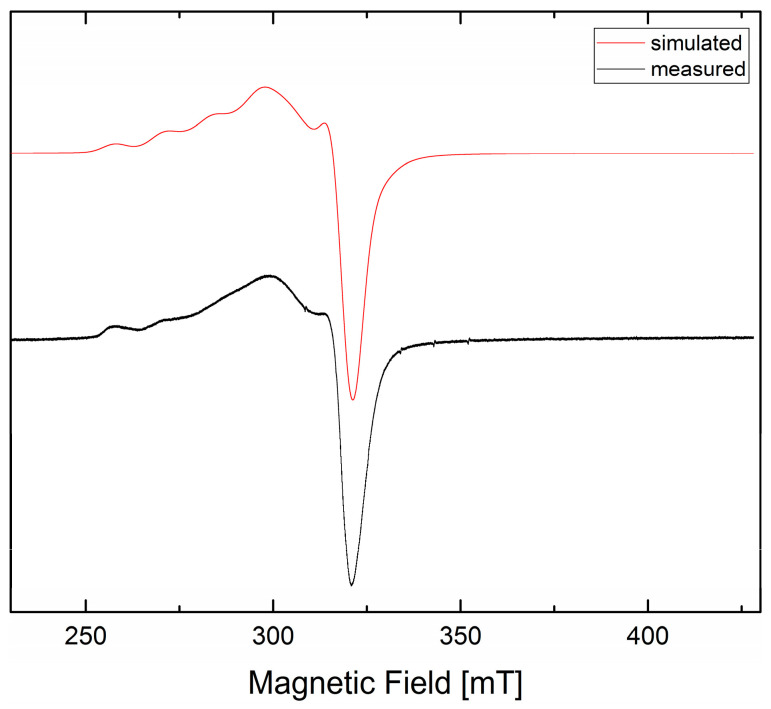
EPR spectrum of complex **2**.

Complex **2** has g-values that are close and comparable to previously reported complexes [39,40,41,42,43,44]. An increase in the ionic character is expected because the g-values (Table 5) are somewhat higher than those of previously reported covalently bound compounds. As g_‖_ > g_⊥_ > g_e_, the copper center is coordinated either in an octahedral fashion or elongated in the z^2^ axis, and the unpaired electron is located in the x^2^-y^2^ orbital (Figure 6). There is a clear alignment between the hyperfine coupling constants and the g-values found.

**Figure 7:**
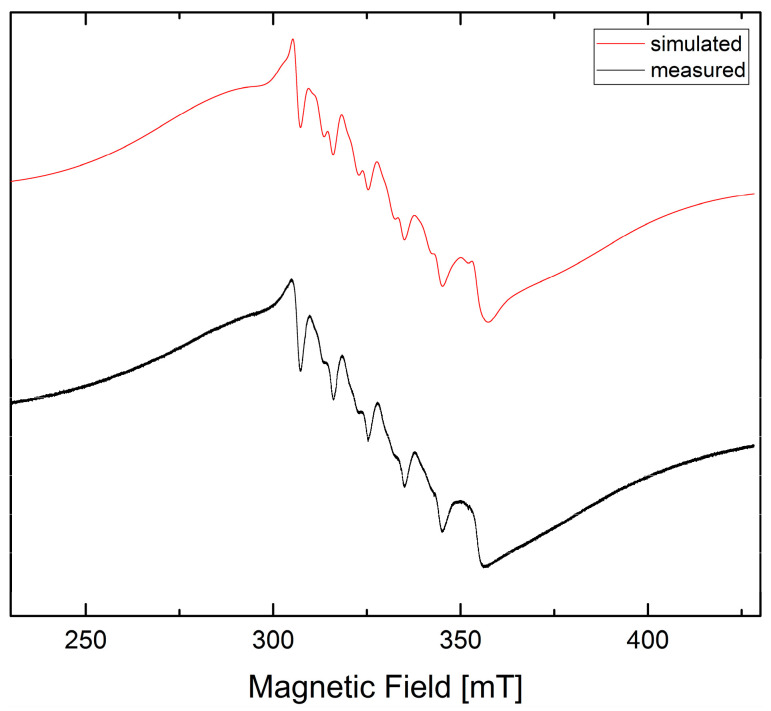
EPR spectrum of complex **5**.

The g-/A- and D-values of complex **5** (Table 6) are determined by applying and resolving the ^55^Mn sextet, assigned to the |−1/2, *m*> to |1/2, *m* > transition. The coordination around the Mn(II) ion is almost *O_h_* (Figure 7). The g-values of this complex are comparable to the previously reported Mn^2+^ complexes, with values close to the free electron (g_e_ = 2.0023). The hyperfine coupling constants are in good accordance with previously described Mn^2+^ complexes [45,46,47].

### 2.7. Antioxidants

Antioxidant substances are described as substances with low concentrations that can inhibit the oxidation of substances. Antioxidant activity plays an essential role in the medical sector by demonstrating a critical effect in exhibiting the risk of chronic diseases such as heart disease and cancer.

The DPPH• free radical scavenging method was used to study the antioxidant activities of both the ligand and complexes **1**–**5** [38]. When the DPPH• absorbance at 517 nm (purple color) decreases, it is possible to determine the scavenging activity spectrophotometrically. Stock solutions of 5 × 10^−4^ M of DPPH, ligand, and its M (II) complexes were prepared in DMF. A total of 400 μL of DPPH solution was added to 500, 1000, 1500, 2000 and 2500 μL of each tested sample. The resulting mixtures were diluted to a total volume of 4.0 mL by DMF to obtain final concentrations of 62.5, 125, 187.5, 250 and 312.5 μM, respectively. The control was prepared by diluting 0.40 mL of 5 × 10^−4^ M DPPH• solution with 3.6 mL of DMF.

**Figure 8:**
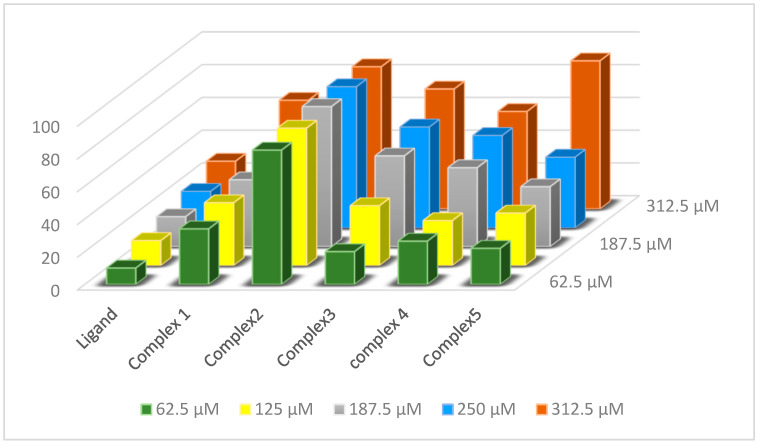
DPPH scavenging activity of ligand and its complexes.

The scavenging activities of the ligand and all complexes depend on concentration, as shown in Table 7 and Figure 8. There is a direct relation between the scavenging activity and concentration, as clearly shown by the results. The ligand has a scavenging activity range between 10.0 and 29.1%, while all M(II) show much higher activities between 20.0 and 90.4%. Complex **5** has the highest scavenging activity while complex **3** shows the lowest due to the positive charge density. This could be attributed to the fact that the lower the charge density, the more stable the positive charge will be. For this reason, complex **3** has shown lower activity than complex **5**.

### 2.8. Antimicrobial Activities

The antibacterial and antifungal properties of the ligand and its metal complexes were examined by the disk diffusion method in vitro against different strains of Gram-negative (*Ec* and *Pm*) and Gram-positive (*Sa* and *Bc*) bacteria, and fungi (*Ca*). The minimum inhibitory concentrations (MICs) of all prepared compounds are illustrated in Table 8. The complexes demonstrate variations in MIC measurements regarding the growth of the bacterial strains tested. The data show that the ligand exhibits low activity against Gram-negative bacteria (*E. coli*) and does not have any against fungal species and Gram-positive bacteria. The activity of the ligand may arise from the presence of both the azomethine and hydroxyl groups [48]. After complexation, there was a great enhancement in activity for complexes. This could be attributed to the chelation theory [49]. Chelation might have reduced the polarity of the metal ions via partial sharing of the positive charge of the transition metal ions with the N and O donor atoms of the ligand and electron delocalization throughout the chelate ring system. This increases the lipophilic character of the metal ions, allowing the complexes to permeate the lipid membrane layers of the bacterial cell wall, which in turn increases the activity of the complexes and limits further microorganism growth [49,50]. Complex **2** shows remarkable activity against Gram-positive bacteria (*B. subtilis*, *S. aureus*), with an MIC value of 8 μg ml^−1^, higher than the two positive controls. It also shows very high activity against Ca fungi with 8 μg ml^−1^, higher than the positive control with Oxytetracycline. Complex **3’s** activity against Gram-positive bacteria *S. aureus* has a value of 8 μg ml^−1^, which is comparable with Oxytetracycline and higher than fluconazole-positive controls. Complex **2** also shows excellent antifungal activity against *C. albicans* with an MIC value of 16 μg ml^−1^. All other complexes show moderate to high antimicrobial activities.

### 2.9. Oxidation of Aniline

**Scheme 2:**
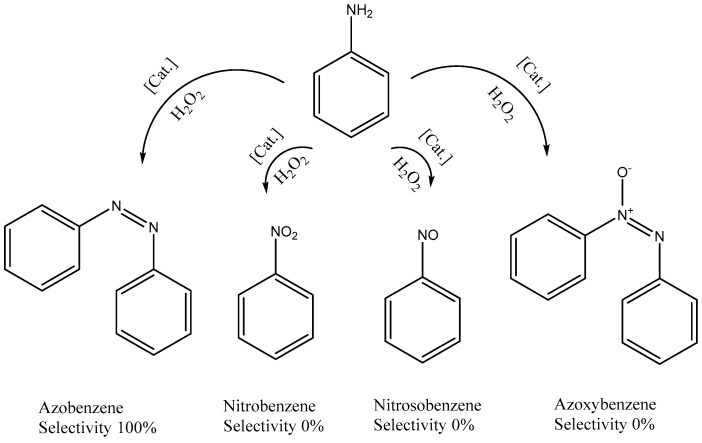
Oxidation of aniline to azobenzene.

M(II) complexes were assessed for their catalytic activities as catalysts for the oxidation of aniline with a catalyst-to-aniline weight ratio of 1:10 using DMF as solvent and 30% H_2_O_2_ as the oxidizing agent at room temperature for one hour. GC–MS is used to manage the reaction. Azobenzene is the single product generated during the reaction in each case (Scheme 2).

Complex **4** was noticed to have the highest activity with a yield of 91.4%, while complex **1** showed the lowest activity with a 15% yield only. The other complexes’ activities ranged between high to moderate activities with yields ranging from 34.0 to 81.4%.

Complex **4** was used to study its catalytic activity toward a different number of substituted anilines. Its activities ranged from 14% to 62%, as shown in Table 9. It seems that the type of substituent on aniline plays a role, depending on whether it is an electron-withdrawing or electron-donating group. In general, the substituted anilines with electron-withdrawing groups showed better activities. The product yield increases as the electron-withdrawing groups’ strength increases [51].

## 3. Experimental Section

### 3.1. Materials and Methods

The chemicals used in the synthesis were utilized without further purification. 2-flurobenzoic hydrazide, salicylaldehyde, M(NO_3_)_2_.XH_2_O, EDTA, xylenol orange, chloroform, ethyl acetate, Dimethyl sulfoxide and DPPH• free radical were all purchased from Sigma Aldrich.

UV–Vis spectra were recorded on UV-1650 PC SHIMADZU spectrophotometer (Shimadzu, Kyoto, Kyoto) in DMF solutions. The infrared data were obtained from SHIMADZU FT-IR instrument in the range of 4000–400 cm^−1^ in KBr pellets. The molar conductivity measurements were conducted using a WTW LF318 conductivity meter equipped with a WTW Teracon 325 (Xylem Inc., Washington, DC, USA) conductivity cell. Molar conductivities were also measured in DMSO (Dimethyl sulfoxide) solution at a concentration of 10^−4^ M and 25 °C. Elemental analyses were performed using a Perkin-Elmer 2400II CHNS-O (Perkin-Elmer, Waltham, MA, USA) elemental analyzer. Metal analyses were conducted through titration using EDTA with xylenol orange as an indicator and acetic acid as a buffer. ^1^H and ^13^C NMR measurements were performed on an NMR BRUKER 400 MHZ (Bruker, Billerica, MA, USA) spectrometer, using DMSO-d_6_ as a solvent. Chemical shifts (δ) were reported in parts per million (ppm) and referenced to tetramethylsilane (TMS, 0.00 ppm) as an internal standard. Thermal analyses were performed at a heating rate of 10 °C/min in the range of R.T. to 800 °C under an inert atmosphere using a PCT-2 A thermo balance analyzer. EPR spectra were recorded on a JEOL JES-FA 200 (JEOL, Akishima, Tokyo) spectrometer. The spectra were measured at a microwave frequency of approximately 9.27 GHz with a microwave power of 5 mW. All samples are prepared in concentration of 10^−4^ mol/L in dried DMSO. Measurements at low temperatures (<283 K) were performed using liquid nitrogen for cooling. GC–MS data were obtained using a Varian Saturn 2000 (Agilent, Santa Clara, CA, USA) ion trap spectrometer interfaced with a Varian GC CP-3800 (Agilent) apparatus using the mode of electronic impact ionization.

### 3.2. Synthesis of Ligand (*L*)

A total of 1.00 g (6.84 mmol) of 2-fluorobenzoic hydrazide was dissolved completely in ethanol in a round-bottom flask with constant stirring at 55 °C. A solution of 0.80 g (6.48 mmol) of salicylaldehyde in ethanol was added in a dropwise manner. The reaction mixture was kept under stirring for two hours. The beige precipitate formed was collected by suction filtration while washing it several times with ethanol. It was dried under vacuum at room temperature. For C_14_H_11_FN_2_O_2_ (258.252 g/mol), the Anal. Found (Calculated) %: C 65.15 (65.11), H 4.36 (4.29), N 10.77 (10.85). FT-IR (cm^−1^): υ(OH): 3300, υ(NH): 3060, υ(C=O): 1660, υ(C=N): 1617. ^1^H NMR (ppm):(N=H): 11.11, (O=H): 12.08, (CH=N): 9.92. ^13^C NMR (ppm): (C=O): 160.20, (C=N): 148.33, Yield (96%).

### 3.3. Synthesis of Complexes

#### 3.3.1. Synthesis of [ZnL_2_(NO_3_)]NO_3_ Complex (**1**)

Dropwise addition of 0.50 g (1.94 mmol) of ligand in chloroform solution to 0.58 g (1.94 mmol) ethyl acetate solution of Zn(NO_3_)_2_.6H_2_O at 55 °C was conducted. The resulting mixture was stirred for two hours. The yellow precipitate solid was separated from the solution by filtration and purified by washing several times with chloroform and ethyl acetate. For C_28_H_22_ZnF_2_N_6_O_10_ (705.89 g/mol), Anal. Found (Calculated) %: C 47.53 (47.64), H 3.17 (3.14), N 11.88 (11.91), Zn 9.15 (9.26). FT-IR (cm^−1^): υ(OH): 3277, υ(NH): 3077, υ(C=O): 1604, υ(C=N):1548, υ(NO_3_^−^):1390. ^1^H NMR (ppm):(N=H): 11.13, (O=H): 12.17, (CH=N): 9.98. ^13^C NMR (ppm):(C=O): 161.16, (C=N): 148.67, Yield 0.77 g (56%).

#### 3.3.2. Synthesis of [CuL_2_(NO_3_)]NO_3_ Complex (**2**)

The complex was synthesized as stated above for complex **1**, using 0.50 g (1.94 mmol) of ligand and 0.47 g (1.94 mmol) of Cu(NO_3_)_2_.3H_2_O. The green precipitate solid was separated from the solution by filtration and purified by washing several times with chloroform and ethyl acetate. For C_28_H_22_CuF_2_N_6_O_10_ (704.06 g/mol), Anal. Found (Calculated) %: C 47.60 (47.77), H 3.21 (3.15), N 12.06 (11.94), Cu 9.22 (9.03). FT-IR (cm^−1^): υ(OH): 3288, υ(NH): 3078, υ(C=O): 1600, υ(C=N): 1551, υ(NO_3_^−^): 1386, Yield 0.94 g (69%).

#### 3.3.3. Synthesis of [CoL_2_(NO_3_)]NO_3_.H_2_O Complex (**3**)

The complex was synthesized as stated above for complex **1**, using 0.50 g (1.94 mmol) of ligand and 0.56 g (1.94 mmol) of Co(NO_3_)_2_.6H_2_O. The brownish-green precipitate solid was separated from the solution by filtration and purified by washing several times with chloroform and ethyl acetate. For C_28_H_24_CoF_2_N_6_O_11_ (717.46 g/mol), Anal. Found (Calculated) %: C 46.69 (46.87), H 3.45 (3.37), N 11.82 (11.71), Co 8.38 (8.21). FT-IR (cm^−1^): υ(OH): 3266, υ(NH): 3067, υ(C=O): 1604, υ(C=N): 1560, υ(NO_3_^−^):1380, Yield 0.69 g (50%).

#### 3.3.4. Synthesis of [NiL_2_(NO_3_)]NO_3_.H_2_O Complex (**4**)

The complex was synthesized as stated above for complex **1**, using 0.50 g (1.94 mmol) of ligand and 0.46 g (1.94 mmol) of Ni(NO_3_)_2_.3H_2_O. The light green precipitate solid was separated from the solution by filtration and purified by washing several times with chloroform and ethyl acetate. For C_28_H_24_NiF_2_N_6_O_11_ (717.22 g/mol). Anal. Found (Calculated) %: C 47.02 (46.89), H 3.32 (3.37), N 11.59 (11.72), Ni 8.14 (8.18). FT-IR (cm^−1^): υ(OH): 3288, υ(NH): 3066, υ(C=O): 1608, υ(C=N): 1555, υ(NO_3_^−^): 1386, Yield 0.95 g (68%).

#### 3.3.5. Synthesis of [MnL_2_(NO_3_)]NO_3_ Complex (**5**)

The complex was synthesized as stated above for complex **1**, using 0.50 g (1.94 mmol) of ligand and 0.49 g (1.94 mmol) of Mn(NO_3_)_2_.4H_2_O. The deep blackish-green precipitate solid was separated from the solution by filtration and purified by washing several times with chloroform and ethyl acetate. For C_28_H_22_MnF_2_N_6_O_10_ (695.45 g/mol), Anal. Found (Calculated) %: C 48.37 (48.36), H 3.13 (3.19), N 12.21 (12.08), Mn 8.05 (7.90). FT-IR (cm^−1^): υ(OH): 3289, υ(NH): 3078, υ(C=O): 1600, υ(C=N): 1537, υ(NO_3_^−^): 1385, Yield 0.77 g (57%).

### 3.4. Antioxidant Activity

The antioxidant activities of both the ligand L and the metal complexes were evaluated using the DPPH (2,2-diphenyl-1-picrylhydrazyl radical) scavenging assay as reported earlier [49]. The scavenging activity of each sample was measured by using the following formula [52]:

Scavenging activity (%) = [(A_0_ − A_s_)/A_0_] × 100% (1)

where A_s_ is the absorbance of the sample, and A_0_ is the absorbance of the DPPH without the sample (control). The data for antioxidant activity are obtained by triplicate determinations as mean ± standard deviation.

### 3.5. Antimicrobial Activity

The antimicrobial activity of the ligand and its complexes was measured using micro-broth dilution minimum inhibition concentration against Gram-negative and Gram-positive bacteria and *Candida albicans* fungus, as previously reported [36,53].

### 3.6. Oxidation of Aniline

To a solution of 8 mmol of aniline, 7.00 mL of dimethylsulfoxide (DMSO), 30% H_2_O_2_ (10 mmol) and complexes **1**–**5** (0.015 mmol) were added separately. The mixture was stirred for 24 h overnight. GC-Ms was used to follow the reaction and to identify the products [54].

## 4. Conclusions

In this work, a Schiff base ligand, 2-fluoro-N’-[(E)-2-hydroxyphenyl] methylene] benzohydrazide and its metal complexes with a general formula [ML_2_ (NO_3_)]NO_3_.nH_2_O, where [M = Co (II), Cu (II), Ni (II), Zn (II), Mn (II)], and [n = 0, 1], were prepared and characterized using different techniques (NMR, EPR, IR, UV-vis, TGA, EA and conductivity). As a result of the analytical and spectral data, the ligand is found to be a bidentate chelate and coordinates to central M(II) ions from the carbonyl oxygen (C=O) and azomethine nitrogen (CH=N) atoms with a stoichiometric ratio of 1:2. The molar conductance data illustrate that one nitrate group is bound to the central metal ion in the coordination sphere while another is in the complexes’ outer coordination sphere. The IR spectral data also show that one of the nitrates groups is coordinated to metal ions bi-dentately in all the investigated complexes. Hence, the coordination number is six for all complexes, as shown in Scheme 1.

DPPH scavenging is concentration-dependent; complex **5** showed the highest activity (90.4%) while Complex **4** showed the lowest (59.5%). The antibacterial activities of the Schiff base and its complexes were examined against various pathogenic bacteria to measure their inhibition potential. Complex **2** showed remarkable activity against Gram (+) bacteria and fungi with an MIC value of 8 μg/mL in comparison with the positive controls, oxytetracycline and fluconazole. The catalytic activities of all complexes were examined in the oxidation of aniline, and the results illustrated that all complexes had a 100% selectivity in producing only azobenzene, with complex **4** having the highest activity (91%).

## Figures and Tables

**Table 1:** Analytical data, molar conductivity and melting point for Schiff base ligand and their complexes.

Complexes	Yield (%)	Found (Calculated) %				Λ_m_ (Ω^−1^cm^−1^mol^−1^)	Decomposition Point/°C
		C	H	N	M		
**Ligand (C_14_H_11_FN_2_O_2_)**	96	65.15 (65.11)	4.36 (4.29)	10.77 (10.85)	-	1.085	166
**[ZnL_2_(NO_3_)]NO_3_**	56	47.53 (47.64)	3.17 (3.14)	11.88 (11.91)	9.15 (9.26)	89.8	300
**[CuL_2_(NO_3_)]NO_3_**	69	47.60 (47.77)	3.21 (3.15)	12.06 (11.94)	9.22 (9.03)	86.3	275
**[CoL_2_(NO_3_)]NO_3_.H_2_O**	50	46.69 (46.87)	3.40 (3.37)	11.82 (11.71)	8.38 (8.21)	87.3	178
**[NiL_2_(NO_3_)]NO_3_.H_2_O**	68	47.02 (46.89)	3.32 (3.37)	11.59 (11.72)	8.14 (8.18)	91.3	220
**[MnL_2_(NO_3_)]NO_3_**	57	48.37 (48.36)	3.13 (3.19)	12.21 (12.08)	8.05 (7.90)	89.0	200

**Table 2:** Major infrared spectra data for ligand and its complexes (cm^−1^).

Complexes	υ(OH)	υ(NH)	υ(C=O)	υ(C=N)	υ(NO_3_^−^)						υ(M-N)	υ(M-O)
					ν_1_	ν_2_	ν_3_	ν_4_	ν_1_–ν_4_	ν_0_		
**Ligand**	3300	3060	1660	1617	-	-	-	-	-	-	-	-
**1**	3277	3077	1604	1548	1470	1100	810	1300	170	1390	565	418
**2**	3288	3078	1600	1551	1440	1102	826	1315	125	1386	547	417
**3**	3266	3067	1604	1560	1460	1090	820	1330	130	1380	560	430
**4**	3288	3066	1608	1555	1466	1093	835	1324	142	1386	561	418
**5**	3289	3078	1600	1537	1457	1100	823	1333	148	1385	553	418

**Table 3:** Electronic spectral data of ligand and its complexes.

Compound	λ_max_ (nm)	Absorbance	Band Assignments
**Ligand**	284.0	0.170	π-π*
	296.0	0.161	π-π*
	325.4	0.125	π-π*
	387.7	0.019	n-π*
**1**	277.1	0.061	π-π*
	303.7	0.140	π-π*
	311.4	0.133	π-π*
	359.0	0.109	**(MLCT)**
**2**	275.7	0.157	π-π*
	300.2	0.171	π-π*
	314.9	0.174	π-π*
	391.2	0.198	**(MLCT)**
**3**	276.4	0.207	π-π*
	295.3	0.176	π-π*
	328.2	0.131	π-π*
	404.5	0.061	**(MLCT)**
**4**	286.2	0.214	π-π*
	333.1	0.144	π-π*
	366.7	0.103	π-π*
	405.2	0.079	**(MLCT)**
**5**	278.5	0.106	π-π*
	319.1	0.119	π-π*
	341.5	0.102	π-π*
	413.6	0.109	**(MLCT)**

**Table 4:** Thermo-grams of Schiff base ligand and M(II) complexes.

Complexes	Stage	DTA/°C	Mass Loss %	Total Mass Loss %
Ligand	I	307.7	87.64	87.64
**1**	I	328.3	30.36	51.32
	II	440.0	9.89	
	III	777.7	11.07	
**2**	I	167.1	8.14	33.67
	II	274.7	25.53	
**3**	I	38.9	14.85	78.20
	II	299.8	38.80	
	III	542.0	16.37	
	IV	733.0	8.18	
**4**	I	72.0	3.29	54.61
	II	227.2	18.87	
	III	334.7	19.30	
	IV	407.2	13.15	
**5**	I	271.4	32.89	55.93
	II	432.3	23.04	

**Table 5:** Spin Hamiltonian parameters and fraction of complex **2**.

	Species 1
**g_iso_**	2.182
**g** **_⊥_**	2.079
**g_‖_**	2.389
**A** **_⊥_**	11.8 MHz
**A_‖_**	424 MHz
**HStrain** **_⊥_**	184 MHz
**HStrain_‖_**	243 MHz
**lwpp**	
**weight**	1

**Table 6:** Spin Hamiltonian parameters and fraction of complex **5**.

	Species 1
**g_iso_**	2.000
**g** **_⊥_**	2.000
**g_‖_**	2.000
**A** **_⊥_**	272 MHz
**A_‖_**	265 MHz
**HStrain** **_⊥_**	145 MHz
**HStrain_‖_**	47.2 MHz
**lwpp**	
**D**	5 × 10^2^ MHz
**E**	1 × 10^2^ MHz
**DStrain**	4 × 10^2^ MHz
**weight**	1

**Table 7:** Antioxidant scavenging activity date of ligand and complexes **1**–**5**.

Complexes	DPPH Scavenging Activity (%)				
Concentration (µM)	62.5	125	187.5	250	312.5
**Ligand**	10.0	15.2	18.3	22.2	29.1
**1**	33.9	38.3	40.8	45.6	66.3
**2**	82.0	83.8	85.5	86.1	86.8
**3**	20.0	36.5	55.4	61.5	73.2
**4**	26.4	27.4	48.2	56.3	59.5
**5**	22.0	32.0	36.6	43.1	90.4

**Table 8:** Minimum inhibitory concentration (MIC, μg ml^−1^) for both complexes against clinical isolates of some bacterial spp. and *Candida albicans*.

Complexes	Gram (−) Bacteria		Gram (+) Bacteria		Fungi
	Ec	Pm	Sa	Bc	Ca
**Ligand**	256	N	N	N	N
**1**	132	N	64	132	64
**2**	64	N	8	8	8
**3**	N	N	8	64	16
**4**	256	N	256	N	N
**5**	132	N	256	132	132
**DMSO (−ve control)**	N	N	N	N	N
**Oxytetracycline (+ve control)**	16	16	8	N	N
**Fluconazole +ve control**	N	N	N	N	8

**Table 9:** Oxidation of aniline derivatives with complex **4** after 4 h.

Reactants	[Complex 4] · H_2_O_2_ 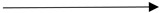 DMSO	Products	Yield
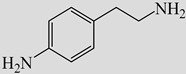	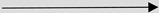	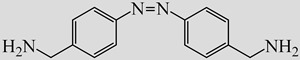	14%
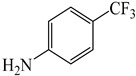	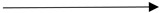	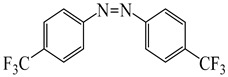	23%
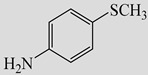	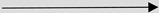	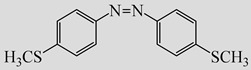	17%
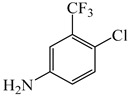	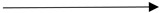	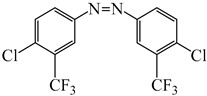	35%
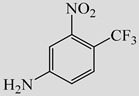	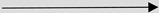	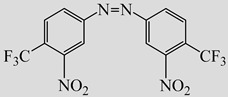	62%
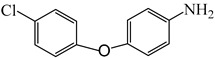	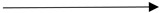	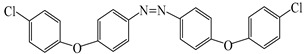	29%

## Data Availability

Data are contained within the article and Supplementary Materials.

## Supplementary Materials

The following supporting information can be downloaded at https://www.mdpi.com/article/10.3390/molecules29235758/s1.
