# Supplementary material for: Synthesis, Characterization and Catalytic/Antimicrobial Activities of Some Transition Metal Complexes Derived from 2-Floro-N-((2-Hydroxyphenyl)Methylene)Benzohydrazide
Source: Molecules. 2024 Dec 5;29(23):5758. doi: 10.3390/molecules29235758 (PMC11643795; doi:10.3390/molecules29235758)
Supplement: Supplementary file 1 [file molecules-29-05758-s001.zip › molecules-3295925-supplementary.pdf]

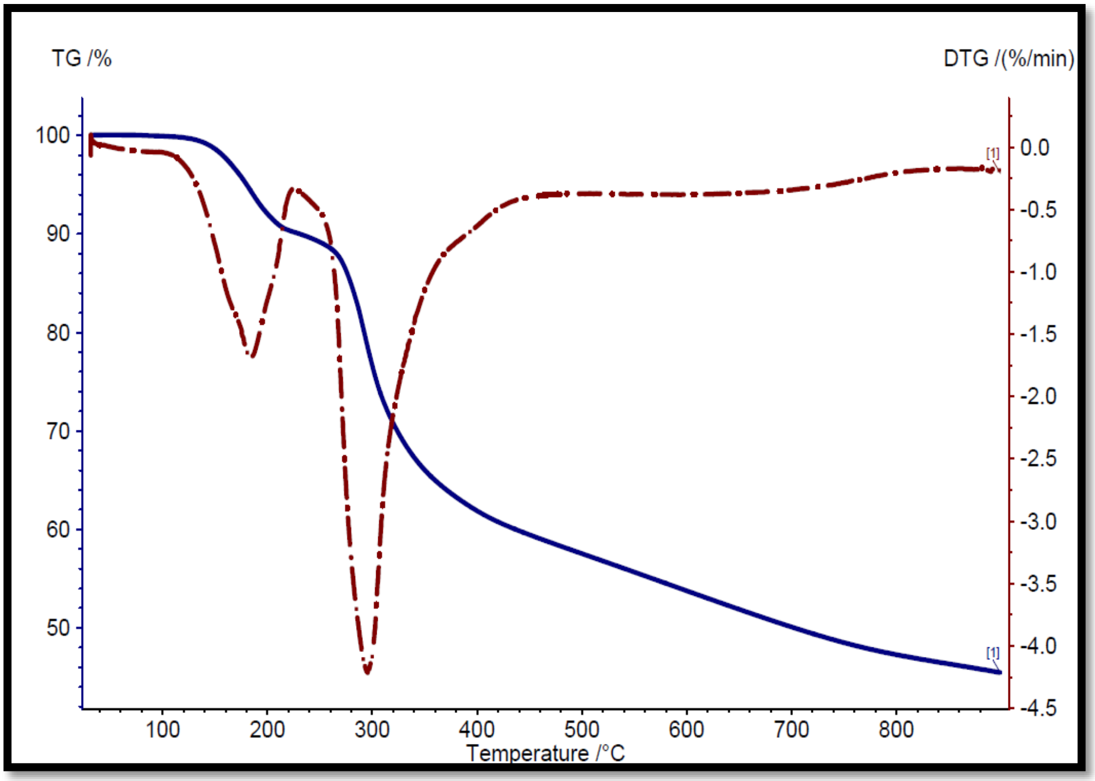

Figure S1:TGA and DTGA of Complex **2**.

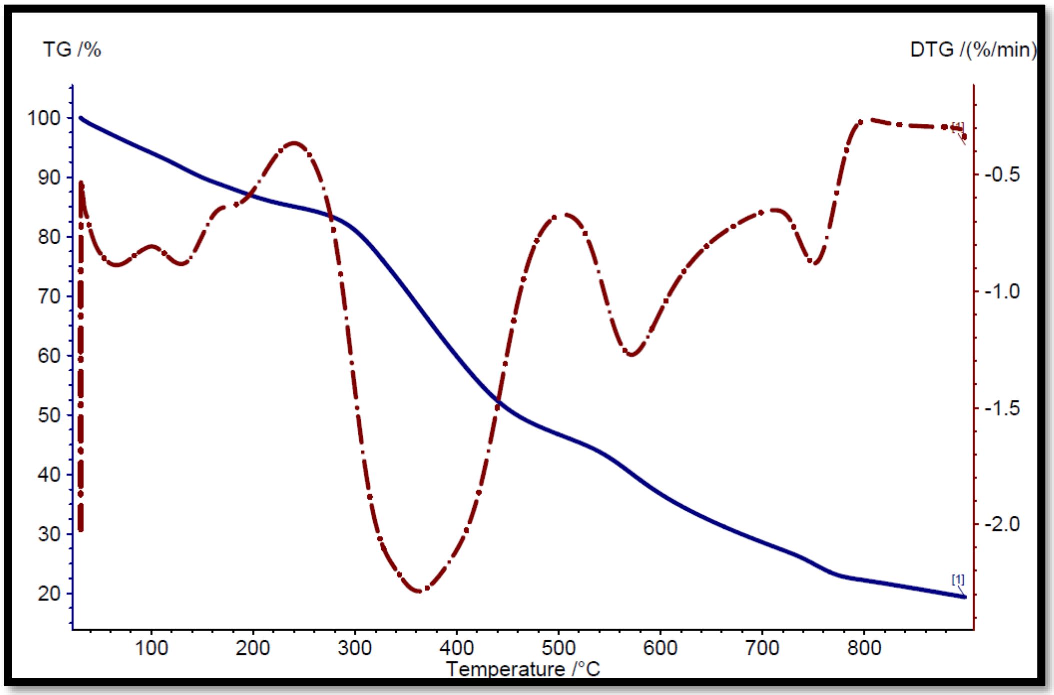

Figure S2: TGA and DTGA of Complex **3**.

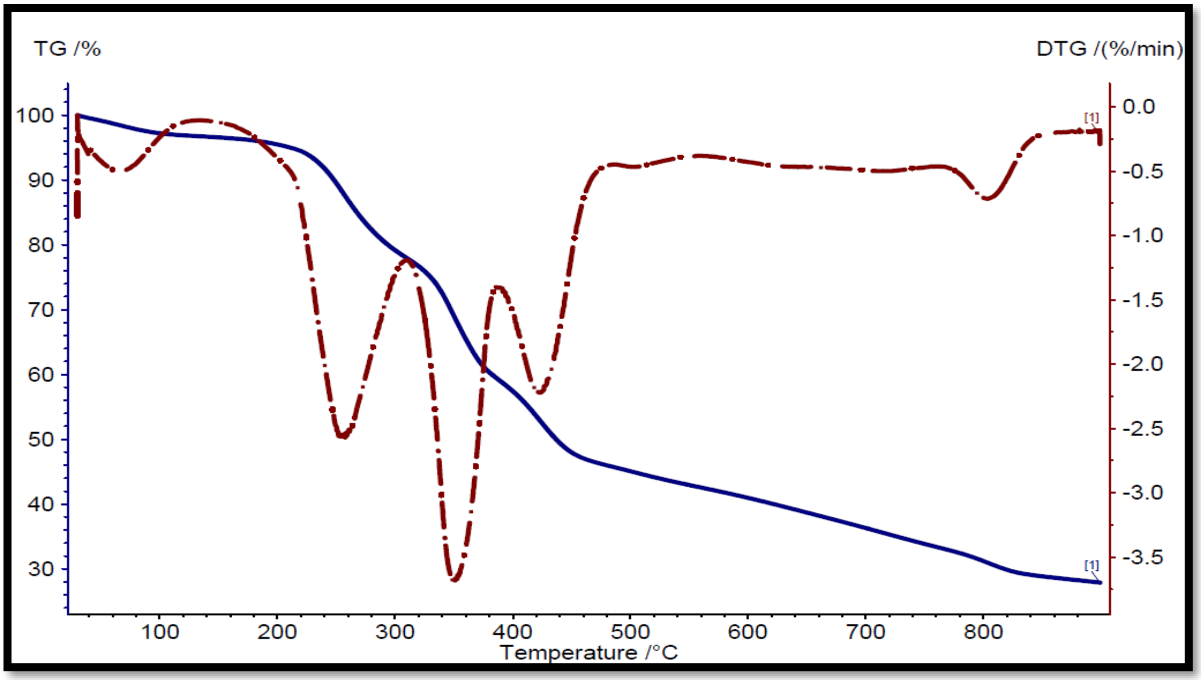

Figure S3: TGA and DTGA of Complex **4**.

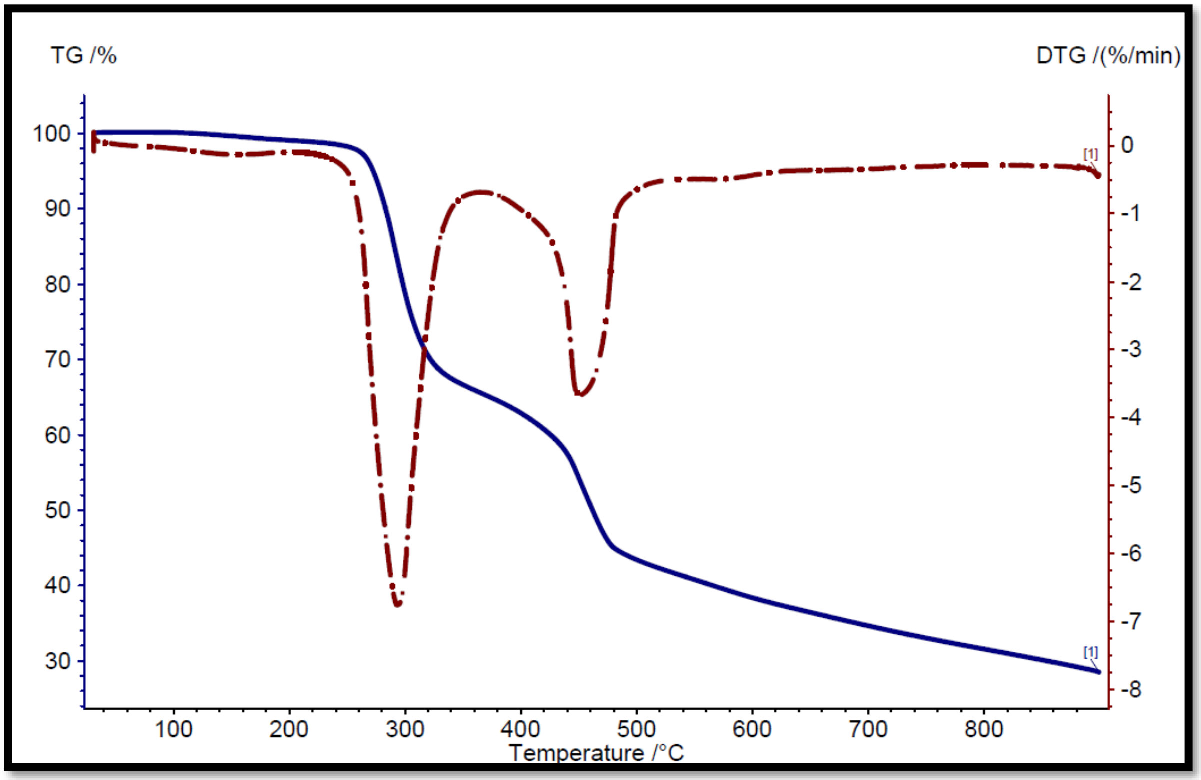

Figure S4: TGA and DTGA of Complex **5**.

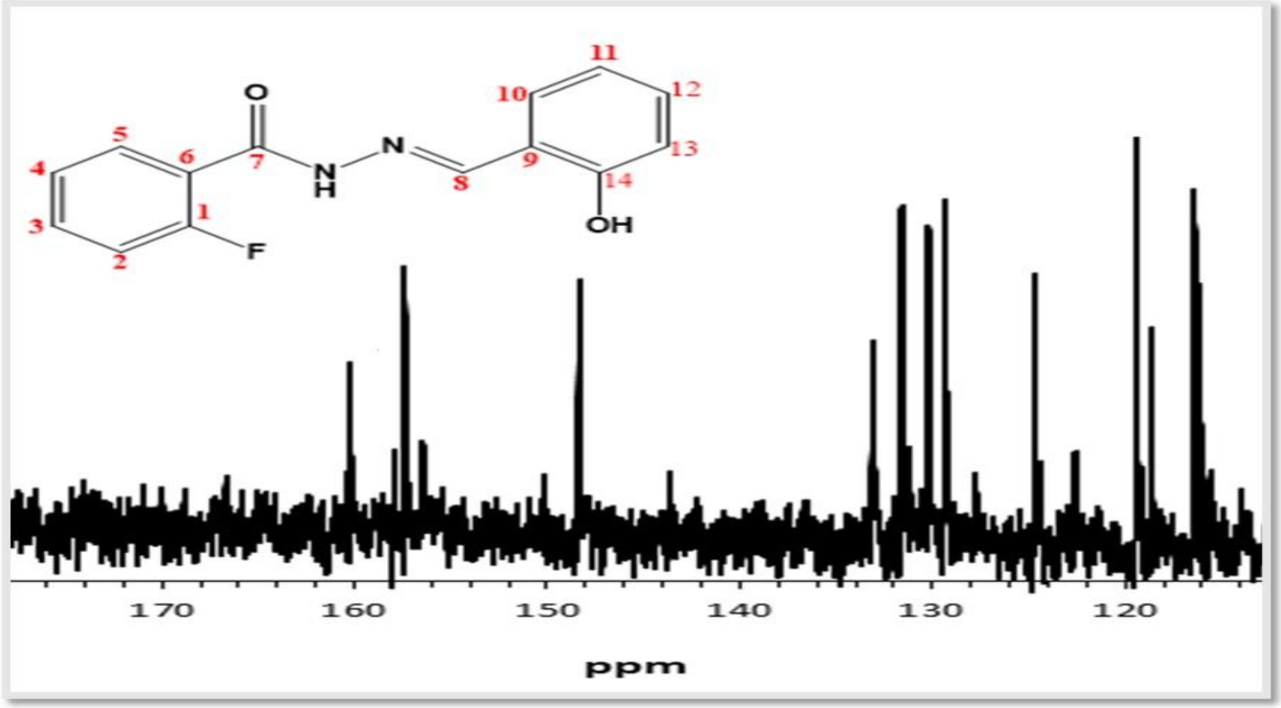

Figure S5: ^{13}C NMR spectrum of the free ligand.

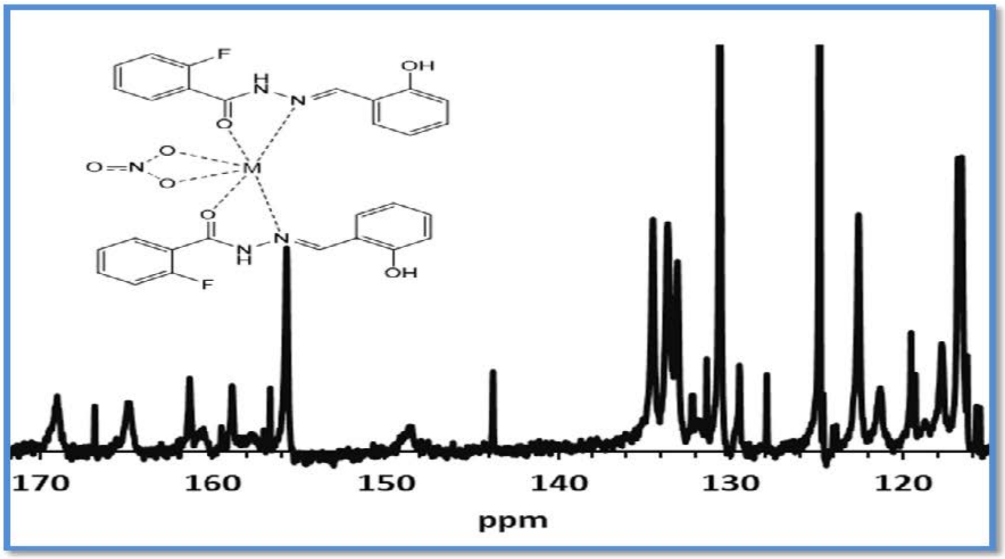

Figure S6: ^{13}C NMR spectrum of complex **1**.
